# Supplementary material for: Performance and Long Distance Data Acquisition via LoRa Technology of a Tubular Plant Microbial Fuel Cell Located in a Paddy Field in West Kalimantan, Indonesia
Source: Sensors (Basel). 2019 Oct 25;19(21):4647. doi: 10.3390/s19214647 (PMC6864700; doi:10.3390/s19214647)
Supplement: Supplementary file 1 [file sensors-19-04647-s001.pdf]

*Supporting Information*

# Performance and Long Distance Data Acquisition via LoRa Technology of a Tubular Plant Microbial Fuel Cell Located in a Paddy Field in West Kalimantan, Indonesia

**Emilius Sudirjo [1,2,*], Pim de Jager [1,3], Cees J.N Buisman[1] and David P.B.T.B. Strik [1,*]**

[1] Environmental Technology, Wageningen University & Research; Bornse Weilanden 9, 6708WG Wageningen, The Netherlands

[2] Government of Landak Regency, West Kalimantan Province, 79357, Indonesia

[3] Plant-e BV, Mansholtlaan 4, 6708PA, Wageningen, The Netherlands

* Correspondence: emilius1.sudirjo@wur.nl or emiliuss@gmail.com (E.S.); david.strik@wur.nl (D.P.B.T.B.S.); Tel.: +31-(0)317-483-447 (D.S.)

**Commented [m1]:** Please carefully check the accuracy of names and affiliations. Changes will not be possible after proofreading.

S1A. Soil was manually hoed about 10-15 cm deep.

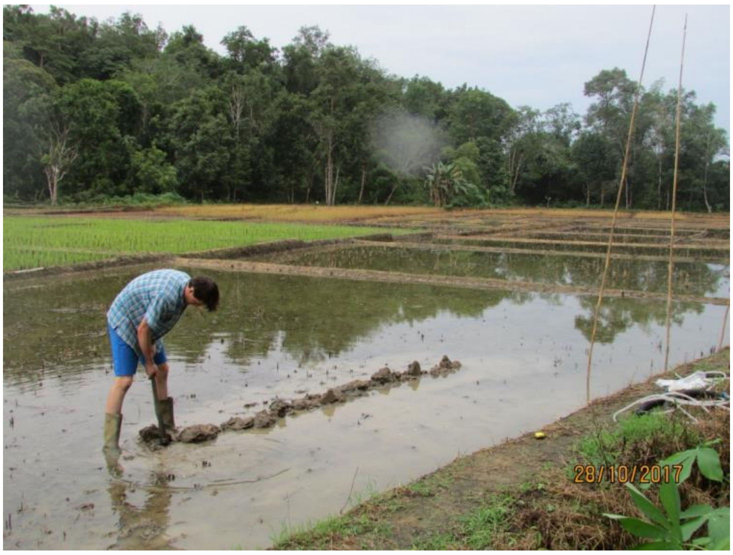

S1B. Tubular Plant-MFCs were installed in the soil by David Strik (left) and Emilius Sudirjo (right). Both silicone tube ends were supported with bamboo sticks.

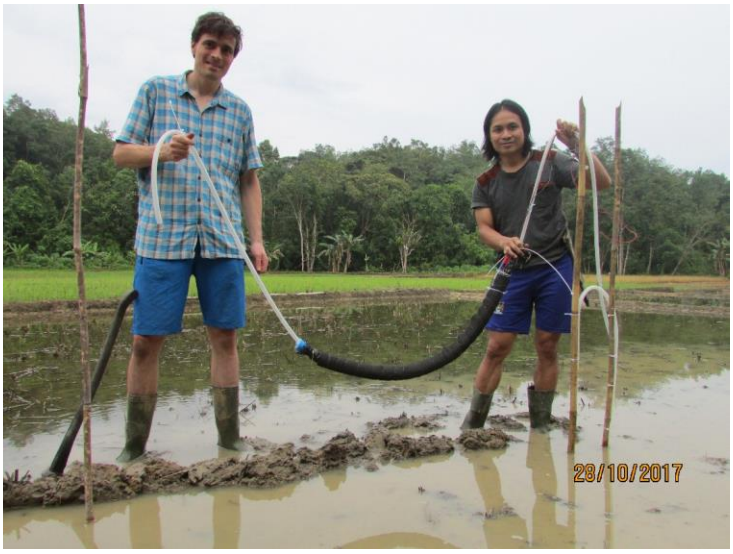


S1C. Plant-MFC after installation. From left to right: Plant-MFC1, Plant-MFC2, Plant-MFC3, and two other Plant-MFCs that are not related to this study.

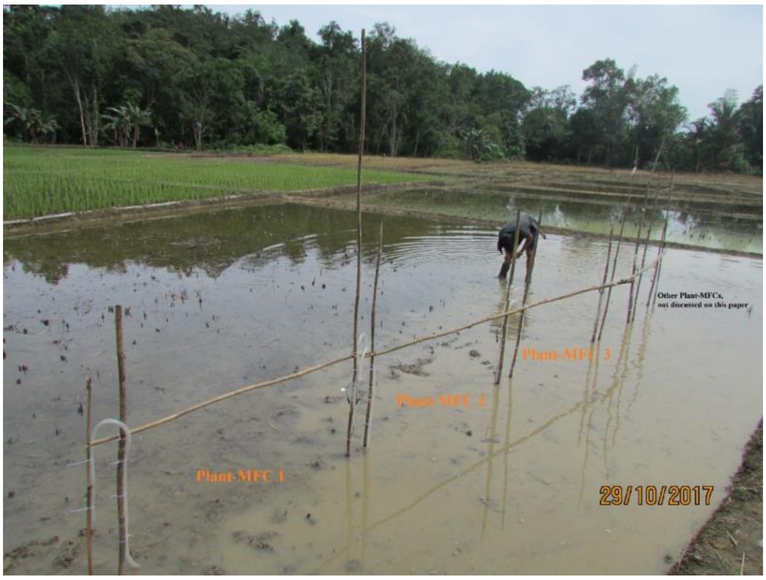


**Figure S1.** Plant-MFC installation in the paddy field. Tububar Plant-MFCs were installed prior to paddy transplantation.

S2A. Harvesting aboveground biomass at one square meter area (marked with bamboo sticks) on top of Plant-MFC1.

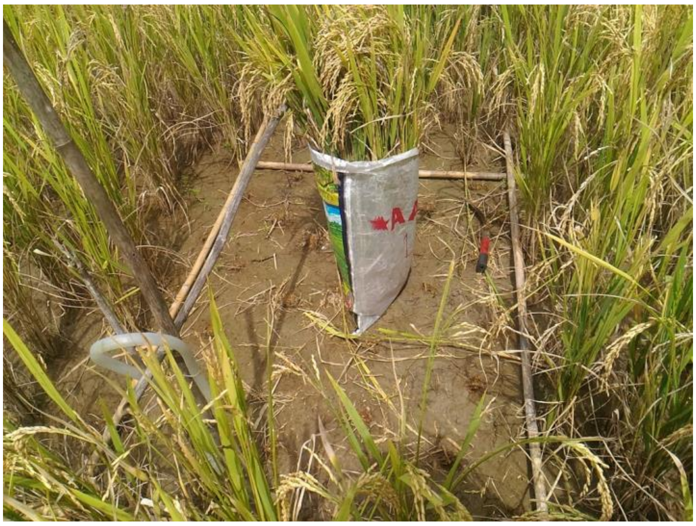

S2B. Plant-MFCs reactor (under soil marked with bamboo sticks) after biomass collection.

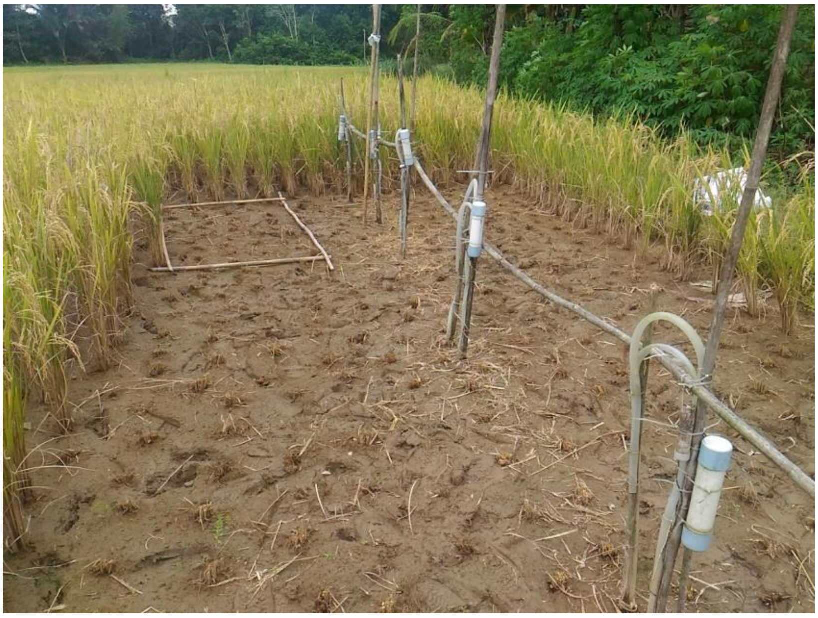

S2C. Nine 1-square meter plots for aboveground biomass collection.

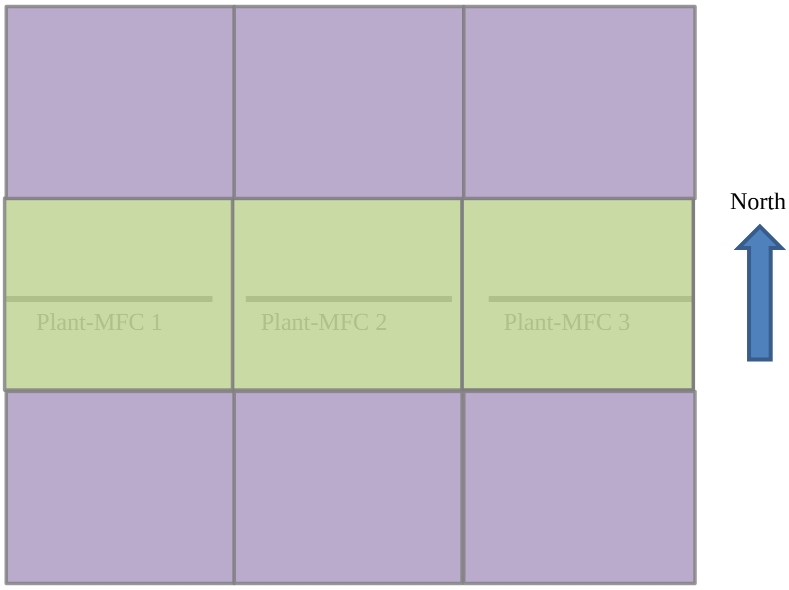


**Figure S2**. Aboveground biomass collection during the third crop season (on 13 February 2019)

S3A. 29 November 2017

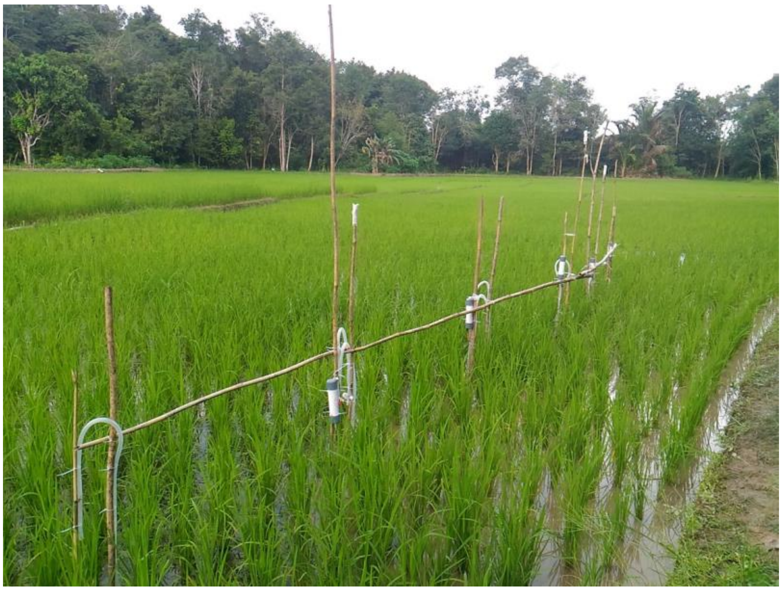

S3B. 30 June 2018

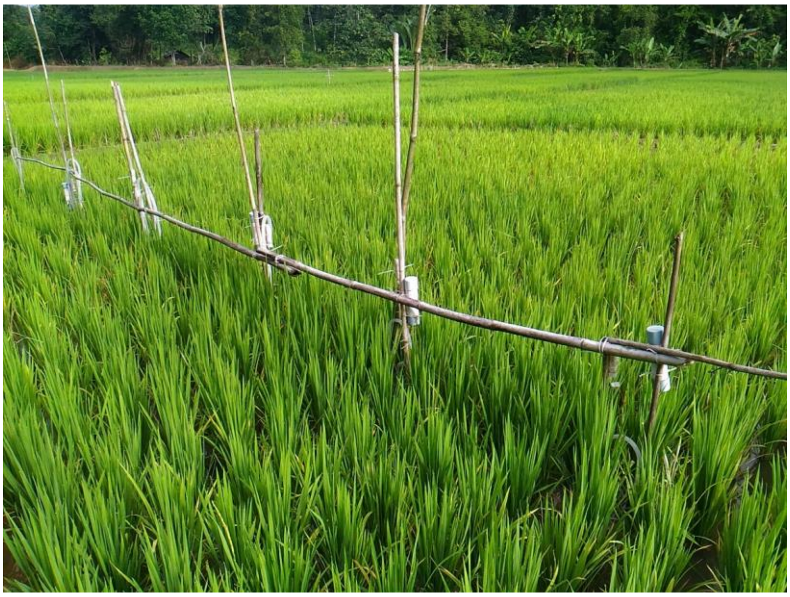

S3C. 17 July 2018

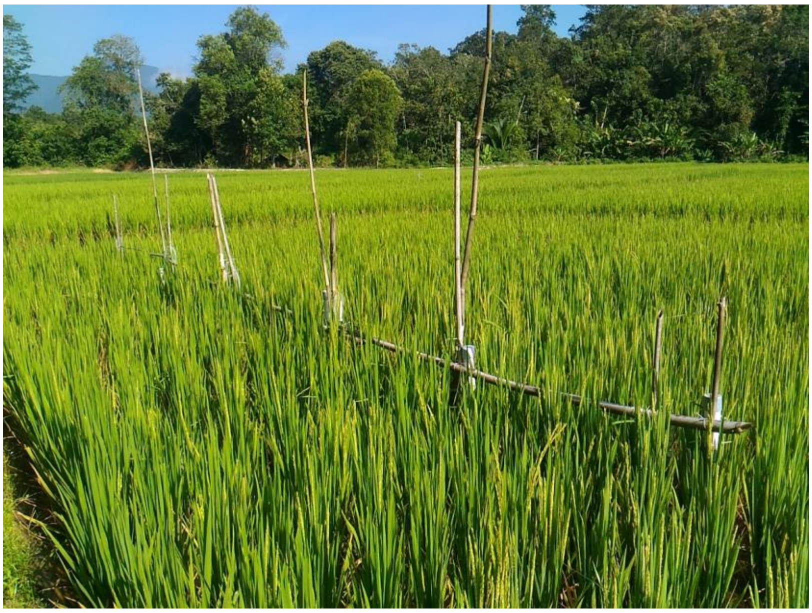

S3D. 13 February 2019 (Plant-MFC1)

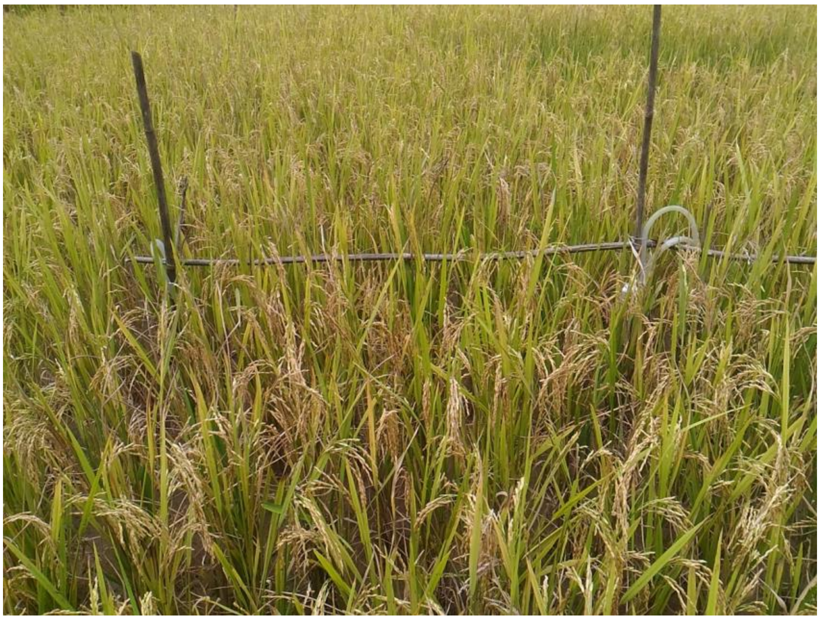

S3E. 13 February 2019 (Plant-MFC2)

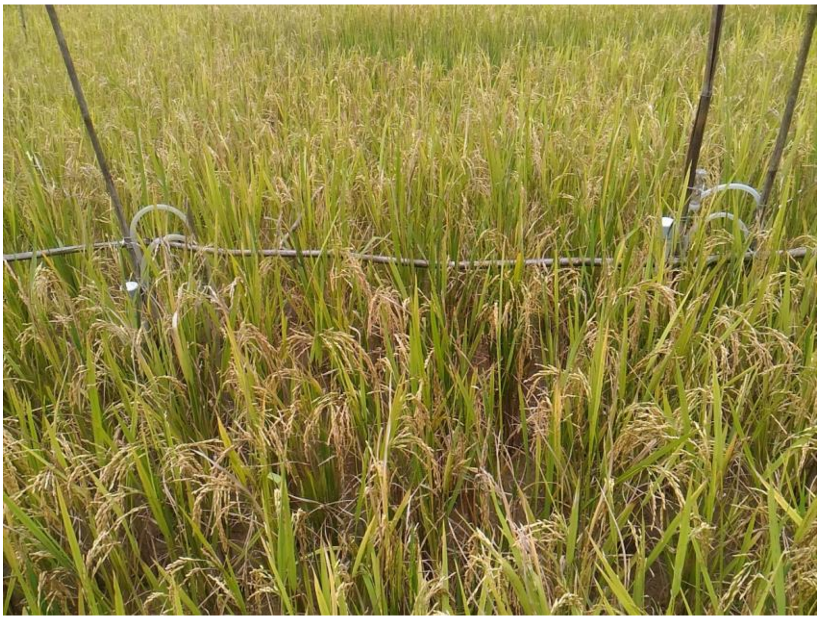

S3F. 13 February 2019 (Plant-MFC31)

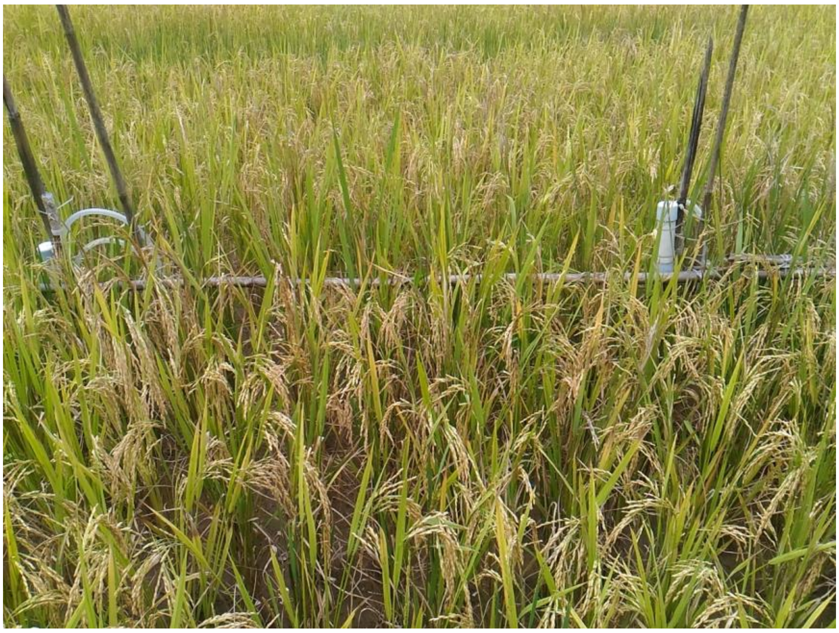

S3G. 5 August 2019 (the LoRa sensor is under the small roof)

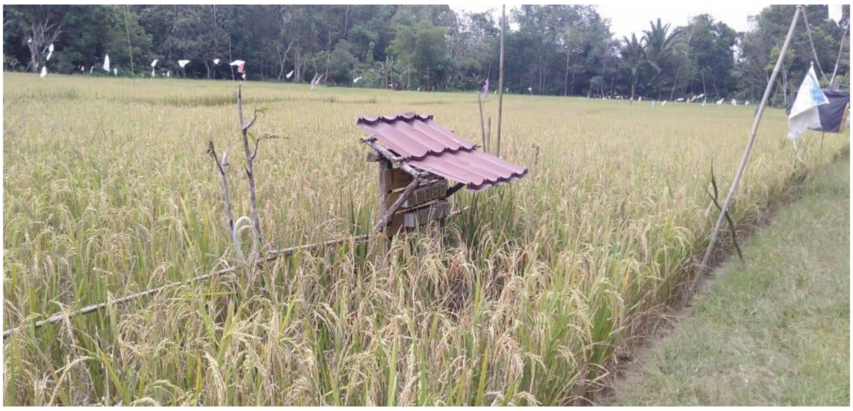

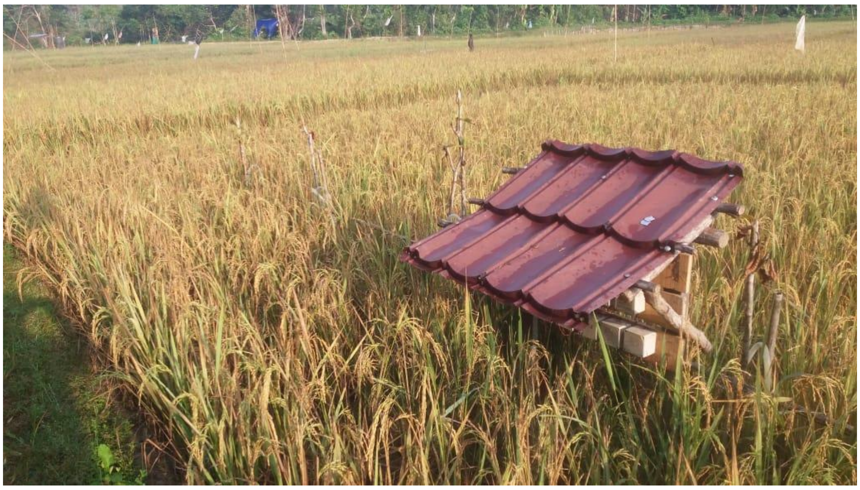

**Figure S3**. Rice paddy visual observations during different stages of growth

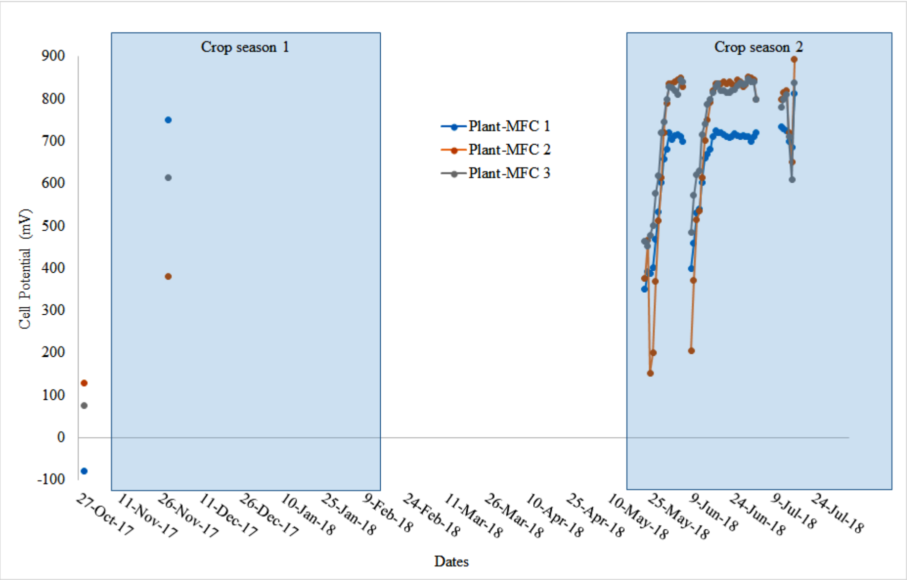


**Figure S4**. Plant-MFC performance manually measured with a multimeter before LoRa data acquisition installation.

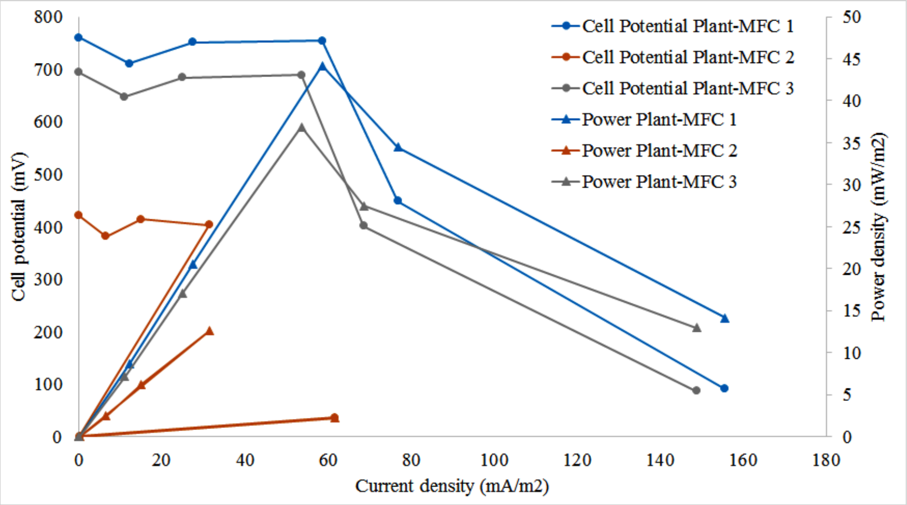


**Figure S5**. Polarizarion curve on 29 November 2017. Current density, cell potential and power density.

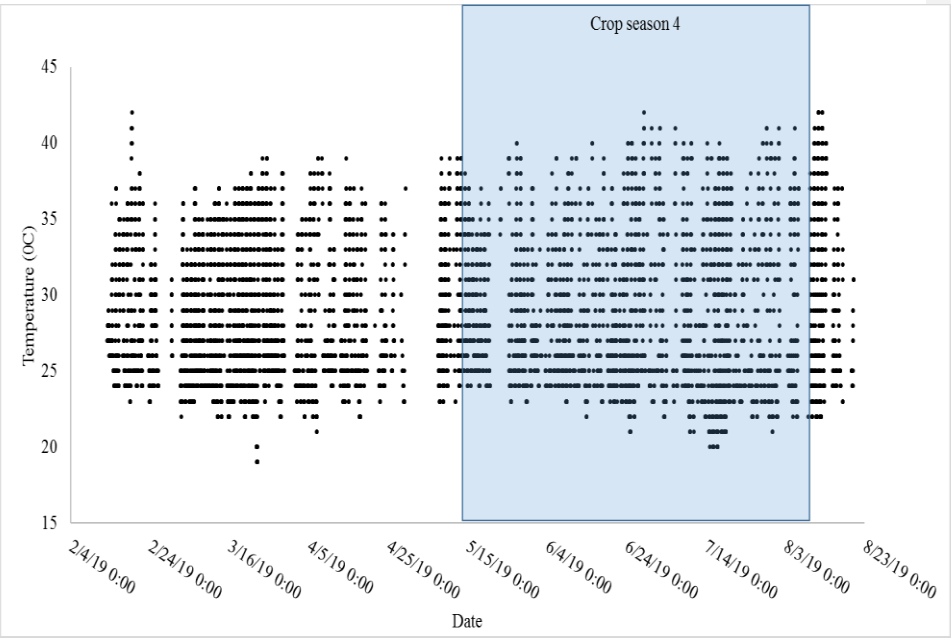


**Figure S6.** Temperature fluctuations during the fourth crop season.

**Table S1.** Microbial Relative abundance for classes, orders, families and genera.

Table S1.A Classes

| Kingdom | Phyla | Classes | Group I (From Mid reactor) | | Group II (end of reactor) | | Group III (Far from reactor) | |
|---|---|---|---|---|---|---|---|---|
| | | | A | C | B | D | F | E |
| k__Bacteria | p__Proteobacteria | c__Betaproteobacteria | 9.75 | 11.67 | 6.38 | 14.03 | 19.35 | 17.60 |
| k__Bacteria | p__Acidobacteria | c__Solibacteres | 7.15 | 7.37 | 8.63 | 8.32 | 9.41 | 7.65 |
| k__Bacteria | p__Nitrospirae | c__Nitrospira | 3.78 | 3.66 | 6.39 | 4.19 | 7.26 | 6.11 |
| k__Bacteria | p__Acidobacteria | c__Acidobacteria-6 | 4.45 | 7.26 | 5.93 | 6.31 | 7.97 | 5.89 |
| k__Bacteria | p__Planctomycetes | c__Planctomycetia | 5.27 | 6.04 | 5.28 | 4.30 | 4.73 | 3.78 |
| k__Bacteria | p__Verrucomicrobia | c__[Methylacidiphilae] | 3.81 | 3.90 | 3.74 | 4.09 | 3.79 | 3.64 |
| k__Bacteria | p__Acidobacteria | c__Acidobacteriia | 5.29 | 4.23 | 5.43 | 4.64 | 3.31 | 3.63 |
| k__Bacteria | p__Planctomycetes | c__Phycisphaerae | 7.08 | 6.89 | 4.42 | 6.48 | 2.75 | 3.34 |
| k__Bacteria | p__Proteobacteria | c__Alphaproteobacteria | 1.76 | 2.52 | 1.61 | 2.12 | 2.86 | 3.32 |
| k__Bacteria | p__AC1 | c__HDBW-WB69 | 0.75 | 1.21 | 0.41 | 0.84 | 2.55 | 2.92 |
| k__Bacteria | p__Planctomycetes | c__BD7-11 | 1.44 | 1.89 | 2.07 | 1.97 | 1.99 | 2.84 |
| k__Bacteria | p__Verrucomicrobia | c__[Pedosphaerae] | 3.89 | 4.06 | 2.74 | 4.64 | 2.20 | 2.74 |
| k__Bacteria | p__Proteobacteria | c__Deltaproteobacteria | 2.54 | 2.54 | 2.41 | 2.86 | 2.69 | 2.47 |
| k__Bacteria | p__Acidobacteria | c__BPC102 | 3.06 | 2.21 | 3.19 | 2.85 | 1.70 | 2.26 |
| k__Bacteria | p__WS3 | c__PRR-12 | 4.10 | 2.90 | 3.89 | 3.35 | 1.40 | 2.25 |
| k__Bacteria | p__Acidobacteria | c__DA052 | 3.03 | 2.41 | 3.81 | 2.42 | 2.05 | 1.99 |
| k__Bacteria | p__OD1 | c__ZB2 | 1.46 | 1.72 | 2.02 | 1.03 | 0.94 | 1.53 |
| k__Bacteria | p__Verrucomicrobia | c__[Spartobacteria] | 0.61 | 0.81 | 0.60 | 0.73 | 1.27 | 1.45 |
| k__Bacteria | p__Chloroflexi | c__Ellin6529 | 3.75 | 2.55 | 1.13 | 2.39 | 0.77 | 1.25 |
| k__Bacteria | p__Chloroflexi | c__Anaerolineae | 3.01 | 2.13 | 3.12 | 1.75 | 0.55 | 1.02 |
| k__Bacteria | p__Firmicutes | c__Clostridia | 0.22 | 0.18 | 0.15 | 0.35 | 0.69 | 1.01 |
| k__Bacteria | p__AD3 | c__ABS-6 | 0.57 | 0.42 | 0.96 | 0.37 | 0.81 | 1.00 |

| | | | | | | | | | |
|---|---|---|---|---|---|---|---|---|---|
| k__Bacteria | p__GAL15 | c__unknown | 0.31 | 0.45 | 1.35 | 0.51 | 1.70 | 0.92 |
| k__Bacteria | p__Chloroflexi | c__Ktedonobacteria | 2.60 | 1.51 | 3.49 | 0.84 | 0.21 | 0.79 |
| k__Bacteria | p__Gemmatimonadetes | c__Gemm-1 | 1.03 | 1.32 | 0.64 | 1.16 | 0.41 | 0.60 |
| k__Bacteria | p__NC10 | c__12-24 | 0.66 | 0.55 | 1.14 | 0.64 | 0.51 | 0.49 |
| | | Others classes <1% | 18.65 | 17.59 | 19.07 | 16.82 | 16.11 | 17.52 |
| | | TOTAL | 100 | 100 | 100 | 100 | 100 | 100 |

Table S1. B Orders

| **Kingdom** | **Phyla** | **Clases** | **Orders** | **Group I (From Mid reactor)** | | **Group II (end of reactor)** | | **Group III (Far from reactor)** | |
|---|---|---|---|---|---|---|---|---|---|
| | | | | A | C | B | D | F | E |
| k__Bacteria | p__Proteobacteria | c__Betaproteobacteria | o__Burkholderiales | 1.687 | 1.197 | 0.828 | 4.738 | 16.184 | 8.949 |
| k__Bacteria | p__Acidobacteria | c__Solibacteres | o__Solibacterales | 6.923 | 7.027 | 8.420 | 7.878 | 9.210 | 7.426 |
| k__Bacteria | p__Nitrospirae | c__Nitrospira | o__Nitrospirales | 3.783 | 3.658 | 6.389 | 4.186 | 7.264 | 6.114 |
| k__Bacteria | p__Acidobacteria | c__Acidobacteria-6 | o__iii1-15 | 3.959 | 6.498 | 5.551 | 5.826 | 7.913 | 5.779 |
| k__Bacteria | p__Proteobacteria | c__Betaproteobacteria | o__Neisseriales | 2.544 | 5.136 | 1.846 | 2.090 | 0.209 | 3.749 |
| k__Bacteria | p__Acidobacteria | c__Acidobacteriia | o__Acidobacteriales | 5.287 | 4.225 | 5.428 | 4.637 | 3.308 | 3.627 |
| k__Bacteria | p__Verrucomicrobia | c__[Methylacidiphilae] | o__S-BQ2-57 | 3.806 | 3.893 | 3.715 | 4.081 | 3.742 | 3.553 |
| k__Bacteria | p__AC1 | c__HDBW-WB69 | o__unknown | 0.752 | 1.210 | 0.412 | 0.838 | 2.552 | 2.924 |
| k__Bacteria | p__Planctomycetes | c__BD7-11 | o__unknown | 1.440 | 1.895 | 2.069 | 1.968 | 1.994 | 2.835 |
| k__Bacteria | p__Verrucomicrobia | c__[Pedosphaerae] | o__[Pedosphaerales] | 3.891 | 4.062 | 2.740 | 4.637 | 2.203 | 2.741 |
| k__Bacteria | p__Proteobacteria | c__Betaproteobacteria | o__Rhodocyclales | 2.896 | 2.944 | 1.755 | 3.055 | 0.815 | 2.678 |
| k__Bacteria | p__Planctomycetes | c__Planctomycetia | o__Gemmatales | 3.756 | 4.285 | 4.152 | 3.249 | 3.431 | 2.608 |
| k__Bacteria | p__Planctomycetes | c__Phycisphaerae | o__WD2101 | 5.201 | 5.020 | 3.408 | 4.857 | 2.128 | 2.439 |
| k__Bacteria | p__WS3 | c__PRR-12 | o__Sediment-1 | 4.077 | 2.886 | 3.878 | 3.342 | 1.399 | 2.251 |
| k__Bacteria | p__Acidobacteria | c__BPC102 | o__MVS-40 | 3.012 | 2.157 | 3.084 | 2.745 | 1.571 | 2.135 |
| k__Bacteria | p__Proteobacteria | c__Alphaproteobacteria | o__Rhodospirillales | 0.767 | 1.348 | 0.881 | 1.103 | 1.260 | 2.038 |

| | | | | | | | | | |
|---|---|---|---|---|---|---|---|---|---|
| k__Bacteria | p__Acidobacteria | c__DA052 | o__Ellin6513 | 3.029 | 2.413 | 3.810 | 2.424 | 2.048 | 1.988 |
| k__Bacteria | p__OD1 | c__ZB2 | o__unknown | 1.456 | 1.722 | 2.020 | 1.030 | 0.943 | 1.531 |
| k__Bacteria | p__Verrucomicrobia | c__[Spartobacteria] | o__[Chthoniobacterales] | 0.608 | 0.809 | 0.603 | 0.734 | 1.265 | 1.451 |
| k__Bacteria | p__Chloroflexi | c__Ellin6529 | o__unknown | 3.746 | 2.545 | 1.126 | 2.389 | 0.772 | 1.254 |
| k__Bacteria | p__Proteobacteria | c__Deltaproteobacteria | o__Myxococcales | 1.481 | 1.480 | 1.096 | 1.353 | 0.734 | 1.108 |
| k__Bacteria | p__AD3 | c__ABS-6 | o__unknown | 0.566 | 0.419 | 0.957 | 0.368 | 0.815 | 1.000 |
| k__Bacteria | p__GAL15 | c__unknown | o__unknown | 0.307 | 0.454 | 1.347 | 0.510 | 1.699 | 0.919 |
| k__Bacteria | p__Chloroflexi | c__Anaerolineae | o__A31 | 2.466 | 1.461 | 2.676 | 1.281 | 0.477 | 0.828 |
| k__Bacteria | p__Proteobacteria | c__Betaproteobacteria | o__Methylophilales | 0.865 | 0.095 | 0.115 | 1.784 | 1.421 | 0.792 |
| k__Bacteria | p__Gemmatimonadetes | c__Gemm-1 | o__unknown | 1.028 | 1.323 | 0.643 | 1.159 | 0.407 | 0.595 |
| k__Bacteria | p__Chloroflexi | c__Ktedonobacteria | o__Thermogemmatisporales | 1.815 | 0.888 | 2.885 | 0.494 | 0.107 | 0.518 |
| k__Bacteria | p__Planctomycetes | c__Phycisphaerae | o__CPla-3 | 1.199 | 1.009 | 0.320 | 0.821 | 0.172 | 0.382 |
| | | | Other orders <1% | 27.655 | 27.939 | 27.848 | 26.423 | 23.957 | 25.785 |
| | | | TOTAL | 100 | 100 | 100 | 100 | 100 | 100 |

Table S1.C Families

| Kingdom | Phyla | Classes | Orders | Families | Group I (from mid-reactor) | | Group II (end of reactor) | | Group III (Far from reactor) | |
|---|---|---|---|---|---|---|---|---|---|---|
| | | | | | A | C | B | D | F | E |
| k__Bacteria | p__Proteobacteria | c__Betaproteobacteria | o__Burkholderiales | f__Comamonadaceae | 1.3 | 0.6 | 0.4 | 4.5 | 15.9 | 8.6 |
| k__Bacteria | p__Acidobacteria | c__Acidobacteria-6 | o__iii1-15 | f__ | 3.6 | 5.9 | 5.3 | 5.3 | 7.6 | 5.4 |
| Unassigned | Other | Other | Other | Other | 5.4 | 5.3 | 5.7 | 4.5 | 4.3 | 5.3 |
| k__Bacteria | p__Acidobacteria | c__Solibacteres | o__Solibacterales | f__ | 2.6 | 2.9 | 3.8 | 3.7 | 4.9 | 3.8 |
| k__Bacteria | p__Proteobacteria | c__Betaproteobacteria | o__Neisseriales | f__Neisseriaceae | 2.5 | 5.1 | 1.8 | 2.1 | 0.2 | 3.7 |
| k__Bacteria | p__Acidobacteria | c__Acidobacteriia | o__Acidobacteriales | f__Koribacteraceae | 5.2 | 4.2 | 5.4 | 4.5 | 3.3 | 3.6 |
| k__Bacteria | p__Verrucomicrobia | c__[Methylacidiphilae] | o__S-BQ2-57 | f__ | 3.8 | 3.9 | 3.7 | 4.1 | 3.7 | 3.6 |

| | | | | | | | | | | |
|---|---|---|---|---|---|---|---|---|---|---|
| k__Bacteria | p__Nitrospirae | c__Nitrospira | o__Nitrospirales | f__0319-6A21 | 1.9 | 1.9 | 3.4 | 1.9 | 4.0 | 3.1 |
| k__Bacteria | p__AC1 | c__HDBW-WB69 | o__ | f__ | 0.8 | 1.2 | 0.4 | 0.8 | 2.6 | 2.9 |
| k__Bacteria | p__Planctomycetes | c__BD7-11 | o__ | f__ | 1.4 | 1.9 | 2.1 | 2.0 | 2.0 | 2.8 |
| k__Bacteria | p__Proteobacteria | c__Betaproteobacteria | o__Rhodocyclales | f__Rhodocyclaceae | 2.9 | 2.9 | 1.8 | 3.1 | 0.8 | 2.7 |
| k__Bacteria | p__Planctomycetes | c__Phycisphaerae | o__WD2101 | f__ | 5.2 | 5.0 | 3.4 | 4.9 | 2.1 | 2.4 |
| k__Bacteria | p__Acidobacteria | c__Solibacteres | o__Solibacterales | f__Solibacteraceae | 3.4 | 3.1 | 3.3 | 3.0 | 2.3 | 2.2 |
| k__Bacteria | p__Acidobacteria | c__BPC102 | o__MVS-40 | f__ | 3.0 | 2.2 | 3.1 | 2.7 | 1.6 | 2.1 |
| k__Bacteria | p__Planctomycetes | c__Planctomycetia | o__Gemmatales | f__Gemmataceae | 2.8 | 3.4 | 3.1 | 2.7 | 3.1 | 2.1 |
| k__Bacteria | p__Proteobacteria | c__Alphaproteobacteria | o__Rhodospirillales | f__Rhodospirillaceae | 0.7 | 1.3 | 0.9 | 1.1 | 1.3 | 2.0 |
| k__Bacteria | p__Acidobacteria | c__DA052 | o__Ellin6513 | f__ | 3.0 | 2.4 | 3.8 | 2.4 | 2.0 | 2.0 |
| k__Bacteria | p__WS3 | c__PRR-12 | o__Sediment-1 | f__PRR-10 | 3.1 | 2.1 | 3.0 | 2.5 | 1.1 | 1.7 |
| k__Bacteria | p__Nitrospirae | c__Nitrospira | o__Nitrospirales | f__[Thermodesulfovibrionaceae] | 1.0 | 0.9 | 1.5 | 1.4 | 1.5 | 1.6 |
| k__Bacteria | p__OD1 | c__ZB2 | o__ | f__ | 1.5 | 1.7 | 2.0 | 1.0 | 0.9 | 1.5 |
| k__Bacteria | p__Verrucomicrobia | c__[Spartobacteria] | o__[Chthoniobacterales] | f__[Chthoniobacteraceae] | 0.6 | 0.8 | 0.6 | 0.7 | 1.3 | 1.5 |
| k__Bacteria | p__Chloroflexi | c__Ellin6529 | o__ | f__ | 3.7 | 2.5 | 1.1 | 2.4 | 0.8 | 1.3 |
| k__Bacteria | p__Verrucomicrobia | c__[Pedosphaerae] | o__[Pedosphaerales] | f__ | 1.2 | 1.2 | 0.9 | 1.8 | 0.8 | 1.2 |
| k__Bacteria | p__AD3 | c__ABS-6 | o__ | f__ | 0.6 | 0.4 | 1.0 | 0.4 | 0.8 | 1.0 |
| k__Bacteria | p__Verrucomicrobia | c__[Pedosphaerae] | o__[Pedosphaerales] | f__auto67_4W | 1.3 | 1.5 | 0.6 | 1.7 | 0.7 | 0.9 |
| k__Bacteria | p__GAL15 | c__ | o__ | f__ | 0.3 | 0.5 | 1.3 | 0.5 | 1.7 | 0.9 |
| k__Bacteria | p__Acidobacteria | c__Solibacteres | o__Solibacterales | f__PAUC26f | 0.5 | 0.5 | 0.9 | 0.6 | 1.5 | 0.9 |
| k__Bacteria | p__Nitrospirae | c__Nitrospira | o__Nitrospirales | f__Nitrospiraceae | 0.3 | 0.5 | 0.8 | 0.3 | 1.1 | 0.9 |
| k__Bacteria | p__Chloroflexi | c__Anaerolineae | o__A31 | f__ | 2.4 | 1.4 | 2.7 | 1.2 | 0.5 | 0.8 |
| k__Bacteria | p__Proteobacteria | c__Betaproteobacteria | o__Methylophilales | f__Methylophilaceae | 0.8 | 0.0 | 0.1 | 1.7 | 1.4 | 0.7 |
| k__Bacteria | p__Gemmatimonadetes | c__Gemm-1 | o__ | f__ | 1.0 | 1.3 | 0.6 | 1.2 | 0.4 | 0.6 |
| k__Bacteria | p__Verrucomicrobia | c__[Pedosphaerae] | o__[Pedosphaerales] | f__Ellin515 | 1.2 | 1.2 | 1.0 | 1.0 | 0.7 | 0.6 |
| k__Bacteria | p__Chloroflexi | c__Ktedonobacteria | o__Thermogemmatisporales | f__Thermogemmatisporaceae | 1.8 | 0.9 | 2.9 | 0.5 | 0.1 | 0.5 |
| k__Bacteria | p__Planctomycetes | c__Planctomycetia | o__Gemmatales | f__Isosphaeraceae | 1.0 | 0.9 | 1.0 | 0.6 | 0.3 | 0.5 |
| k__Bacteria | p__Proteobacteria | c__Betaproteobacteria | o__MND1 | f__ | 0.8 | 0.9 | 0.7 | 1.0 | 0.3 | 0.5 |

| | | | | | | | | | | |
|---|---|---|---|---|---|---|---|---|---|---|
| k__Bacteria | p__Planctomycetes | c__Phycisphaerae | o__CPla-3 | f__ | 1.2 | 1.0 | 0.3 | 0.8 | 0.2 | 0.4 |
| | | | | Other Families <1% | 26.0 | 26.5 | 25.4 | 25.3 | 22.2 | 23.6 |
| | | | | TOTAL | 100 | 100 | 100 | 100 | 100 | 100 |

Table S1. D Genera

| **Kingdom** | **Phyla** | **Classes** | **Orders** | **Families** | **Genera** | **Group I (From Mid reactor)** | | **Group II (end of reactor)** | | **Group III (Far from reactor)** | |
|---|---|---|---|---|---|---|---|---|---|---|---|
| | | | | | | A | C | B | D | F | E |
| k__Bacteria | p__Acidobacteria | c__Acidobacteria-6 | o__iii1-15 | f__ | g_unknown | 3.60 | 5.87 | 5.35 | 5.29 | 7.61 | 5.44 |
| k__Bacteria | p__Proteobacteria | c__Betaproteobacteria | o__Burkholderiales | f__Comamonadaceae | g__unknown | 1.00 | 0.37 | 0.34 | 0.93 | 1.75 | 4.06 |
| k__Bacteria | p__Acidobacteria | c__Solibacteres | o__Solibacterales | f__ | g_unknown | 2.59 | 2.89 | 3.76 | 3.68 | 4.92 | 3.81 |
| k__Bacteria | p__Verrucomicrobia | c__[Methylacidiphilae] | o__S-BQ2-57 | f__ | g_unknown | 3.81 | 3.89 | 3.72 | 4.08 | 3.74 | 3.55 |
| k__Bacteria | p__Nitrospirae | c__Nitrospira | o__Nitrospirales | f__0319-6A21 | g_unknown | 1.86 | 1.88 | 3.38 | 1.93 | 3.98 | 3.13 |
| k__Bacteria | p__AC1 | c__HDBW-WB69 | o__ | f__ | g_unknown | 0.75 | 1.21 | 0.41 | 0.84 | 2.55 | 2.92 |
| k__Bacteria | p__Planctomycetes | c__BD7-11 | o__ | f__ | g_unknown | 1.44 | 1.89 | 2.07 | 1.97 | 1.99 | 2.84 |
| k__Bacteria | p__Proteobacteria | c__Betaproteobacteria | o__Burkholderiales | f__Comamonadaceae | g__Hydrogenophaga | 0.21 | 0.11 | 0.03 | 3.00 | 12.38 | 2.80 |
| k__Bacteria | p__Acidobacteria | c__Acidobacteriia | o__Acidobacteriales | f__Koribacteraceae | g_unknown | 4.54 | 3.49 | 4.61 | 3.61 | 2.47 | 2.64 |
| k__Bacteria | p__Planctomycetes | c__Phycisphaerae | o__WD2101 | f__ | g_unknown | 5.20 | 5.02 | 3.41 | 4.86 | 2.13 | 2.44 |
| k__Bacteria | p__Acidobacteria | c__Solibacteres | o__Solibacterales | f__Solibacteraceae | g__Candidatus Solibacter | 3.35 | 3.06 | 3.26 | 2.98 | 2.32 | 2.22 |
| k__Bacteria | p__Acidobacteria | c__BPC102 | o__MVS-40 | f__ | g_unknown | 3.01 | 2.16 | 3.08 | 2.75 | 1.57 | 2.13 |
| k__Bacteria | p__Proteobacteria | c__Alphaproteobacteria | o__Rhodospirillales | f__Rhodospirillaceae | g_unknown | 0.71 | 1.30 | 0.86 | 1.04 | 1.16 | 2.01 |
| k__Bacteria | p__Acidobacteria | c__DA052 | o__Ellin6513 | f__ | g_unknown | 3.03 | 2.41 | 3.81 | 2.42 | 2.05 | 1.99 |
| k__Bacteria | p__Proteobacteria | c__Betaproteobacteria | o__Neisseriales | f__Neisseriaceae | g__Vogesella | 0.75 | 1.03 | 0.60 | 0.38 | 0.10 | 1.91 |

| | | | | | | | | | | | |
|---|---|---|---|---|---|---|---|---|---|---|---|
| k__Bacteria | p__Planctomycetes | c__Planctomycetia | o__Gemmatales | f__Gemmataceae | g_unknown | 2.47 | 2.84 | 3.04 | 2.42 | 2.96 | 1.87 |
| k__Bacteria | p__WS3 | c__PRR-12 | o__Sediment-1 | f__PRR-10 | g_unknown | 3.11 | 2.10 | 3.02 | 2.50 | 1.10 | 1.70 |
| k__Bacteria | p__Proteobacteria | c__Betaproteobacteria | o__Neisseriales | f__Neisseriaceae | Other | 1.63 | 3.77 | 1.13 | 1.57 | 0.11 | 1.55 |
| k__Bacteria | p__OD1 | c__ZB2 | o__ | f__ | g_unknown | 1.46 | 1.72 | 2.02 | 1.03 | 0.94 | 1.53 |
| k__Bacteria | p__Chloroflexi | c__Ellin6529 | o__ | f__ | g_unknown | 3.75 | 2.55 | 1.13 | 2.39 | 0.77 | 1.25 |
| k__Bacteria | p__Proteobacteria | c__Betaproteobacteria | o__Rhodocyclales | f__Rhodocyclaceae | g_unknown | 2.30 | 2.45 | 1.52 | 2.43 | 0.40 | 1.22 |
| k__Bacteria | p__Verrucomicrobia | c__[Pedosphaerae] | o__[Pedosphaerales] | f__ | g_unknown | 1.24 | 1.21 | 0.94 | 1.83 | 0.79 | 1.16 |
| k__Bacteria | p__AD3 | c__ABS-6 | o__ | f__ | g_unknown | 0.57 | 0.42 | 0.96 | 0.37 | 0.81 | 1.00 |
| k__Bacteria | p__Verrucomicrobia | c__[Pedosphaerae] | o__[Pedosphaerales] | f__auto67_4W | g_uknown | 1.29 | 1.50 | 0.63 | 1.70 | 0.69 | 0.93 |
| k__Bacteria | p__GAL15 | c__ | o__ | f__ | g_unknown | 0.31 | 0.45 | 1.35 | 0.51 | 1.70 | 0.92 |
| k__Bacteria | p__Acidobacteria | c__Solibacteres | o__Solibacterales | f__PAUC26f | g__ | 0.46 | 0.47 | 0.94 | 0.65 | 1.49 | 0.85 |
| k__Bacteria | p__Chloroflexi | c__Anaerolineae | o__A31 | f__ | g_unknown | 2.42 | 1.44 | 2.66 | 1.24 | 0.47 | 0.81 |
| k__Bacteria | p__Proteobacteria | c__Betaproteobacteria | o__Methylophilales | f__Methylophilaceae | g__ | 0.80 | 0.05 | 0.10 | 1.69 | 1.38 | 0.74 |
| k__Bacteria | p__Gemmatimonadetes | c__Gemm-1 | o__ | f__ | g_unknown | 1.03 | 1.32 | 0.64 | 1.16 | 0.41 | 0.60 |
| k__Bacteria | p__Verrucomicrobia | c__[Pedosphaerae] | o__[Pedosphaerales] | f__Ellin515 | g__ | 1.24 | 1.22 | 1.05 | 1.02 | 0.66 | 0.58 |
| k__Bacteria | p__Chloroflexi | c__Ktedonobacteria | o__Thermogemmatisporales | f__Thermogemmatisporaceae | g__ | 1.81 | 0.89 | 2.88 | 0.49 | 0.11 | 0.52 |
| k__Bacteria | p__Planctomycetes | c__Planctomycetia | o__Gemmatales | f__Isosphaeraceae | g__ | 0.97 | 0.89 | 1.01 | 0.56 | 0.31 | 0.49 |
| k__Bacteria | p__Planctomycetes | c__Phycisphaerae | o__CPla-3 | f__ | g_unknown | 1.20 | 1.01 | 0.32 | 0.82 | 0.17 | 0.38 |
| Unassigned | Other | Other | Other | Other | Unassigned_Other | 5.44 | 5.31 | 5.69 | 4.55 | 4.28 | 5.30 |
| | | | | | Other genera <1% | 30.65 | 31.81 | 30.28 | 31.31 | 29.74 | 32.72 |
| | | | | | TOTAL | 100 | 100 | 100 | 100 | 100 | 100 |

**Table S2.** Rainfall data vs peak at Plant-MFC during dry period between crop season 3 and 4

| Date | Mempawah climatology station | | | Paloh climatology data | | | Rain possibility in the research site |
|---|---|---|---|---|---|---|---|
| | Rain (mm) | Wind direction | rain states | Rain (mm) | Wind Direction | Rain states | |
| 14-Feb-19 | 2.80 | NE | moderate rain | 0 | C | no rain | High possibility |
| 15-Feb-19 | 4.00 | C | moderate rain | 0.1 | C | drizzle | Low possibility |
| 16-Feb-19 | 21.50 | NE | heavy rain | 8888 | na | data not available | High possibility |
| 17-Feb-19 | 14.30 | C | heavy rain | 1.8 | C | drizzle | Low possibility |
| 18-Feb-19 | 8888 | C | data not available | 0 | C | no rain | unknown |
| 19-Feb-19 | 0.00 | E | no rain | 0 | C | no rain | unknown |
| 20-Feb-19 | 0.00 | NE | no rain | 0 | C | no rain | unknown |
| 21-Feb-19 | 1.80 | C | drizzle | 0 | C | no rain | unknown |
| 22-Feb-19 | 113.00 | E | storm | 0 | N | no rain | High possibility |
| 23-Feb-19 | 0.00 | NE | no rain | 2.5 | C | moderate rain | Low possibility |
| 24-Feb-19 | 98.10 | NE | storm | 66.7 | C | storm | High possibility |
| 25-Feb-19 | 3.10 | C | moderate rain | 8888 | na | data not available | Low possibility |
| 26-Feb-19 | 9.30 | E | moderate rain | 8888 | na | data not available | Low possibility |
| 27-Feb-19 | 11.2 | NE | heavy rain | 3.4 | C | moderate rain | High possibility |

| | | | | | | | |
|---|---|---|---|---|---|---|---|
| 28-Feb-19 | 3.3 | NE | moderate rain | 8888 | C | data not available | High possibility |
| 1-Mar-19 | 10 | C | heavy rain | 0 | C | no rain | Low possibility |
| 2-Mar-19 | 1.5 | W | drizzle | 0 | C | no rain | unknown |
| 3-Mar-19 | 0 | C | no rain | 14 | C | heavy rain | unknown |
| 4-Mar-19 | 0 | C | no rain | 1.5 | C | drizzle | unknown |
| 5-Mar-19 | 0 | C | no rain | 0 | C | no rain | unknown |
| 6-Mar-19 | 0.2 | C | drizzle | 4.9 | C | moderate rain | unknown |
| 7-Mar-19 | 0 | E | no rain | 0.4 | C | drizzle | unknown |
| 8-Mar-19 | na | W | data not available | 8888 | na | data not available | unknown |
| 9-Mar-19 | 2.50 | C | moderate rain | 0 | C | no rain | Low possibility |
| 10-Mar-19 | 0.00 | W | no rain | 0.5 | N | drizzle | unknown |
| 11-Mar-19 | na | C | data not available | 4.7 | C | moderate rain | unknown |
| 12-Mar-19 | na | na | data not available | 8888 | C | data not available | unknown |
| 13-Mar-19 | 0 | W | no rain | 0 | C | no rain | unknown |
| 14-Mar-19 | 0 | C | no rain | 0 | C | no rain | unknown |
| 15-Mar-19 | 1 | NE | drizzle | 0 | C | no rain | unknown |
| 16-Mar-19 | 18.8 | C | heavy rain | 25.1 | C | heavy rain | High possibility |
| 17-Mar-19 | 0 | NE | no rain | 8888 | C | data not available | unknown |
| 18-Mar-19 | 8888 | na | data not available | 0 | C | no rain | unknown |
| 19-Mar-19 | 0 | C | no rain | 0 | C | no rain | unknown |
| 20-Mar-19 | 0 | W | no rain | 0 | C | no rain | unknown |
| 21-Mar-19 | 0 | SW | no rain | 0 | C | no rain | unknown |
| 22-Mar-19 | 8888 | na | data not available | 0 | C | no rain | unknown |
| 23-Mar-19 | 0 | W | no rain | 8888 | na | data not available | unknown |
| 24-Mar-19 | 0 | W | no rain | 0 | C | no rain | unknown |
| 25-Mar-19 | 0 | SW | no rain | 8888 | N | data not available | unknown |
| 26-Mar-19 | 0 | NE | no rain | 0 | C | no rain | unknown |
| 27-Mar-19 | 0.4 | W | drizzle | 0 | C | no rain | High possibility |
| 28-Mar-19 | 11.4 | SW | heavy rain | 8888 | na | data not available | High possibility |

| | | | | | | | |
|---|---|---|---|---|---|---|---|
| 29-Mar-19 | 1.3 | SW | drizzle | 3.8 | C | moderate rain | High possibility |
| 30-Mar-19 | 0 | SW | no rain | 0 | C | no rain | unknown |
| 31-Mar-19 | 0 | W | no rain | 0 | C | no rain | unknown |
| 1-Apr-19 | 8888 | W | data not available | 0 | C | no rain | unknown |
| 2-Apr-19 | 8888 | na | data not available | 8888 | C | data not available | unknown |
| 3-Apr-19 | 8888 | na | data not available | 8888 | na | data not available | unknown |
| 4-Apr-19 | 8888 | na | data not available | 8888 | na | data not available | unknown |
| 5-Apr-19 | 0 | C | no rain | 0 | C | no rain | unknown |
| 6-Apr-19 | 0 | W | no rain | 0 | C | no rain | unknown |
| 7-Apr-19 | 0 | C | no rain | 0 | N | no rain | unknown |
| 8-Apr-19 | 0 | W | no rain | 0 | C | no rain | unknown |
| 9-Apr-19 | 8888 | na | data not available | 0 | C | no rain | unknown |
| 10-Apr-19 | 8888 | na | data not available | 13.9 | C | heavy rain | High possibility |
| 11-Apr-19 | 0.4 | C | drizzle | 25.7 | E | heavy rain | High possibility |
| 12-Apr-19 | 1.5 | SW | drizzle | 1.8 | C | drizzle | unknown |
| 13-Apr-19 | 66.4 | C | storm | 0.1 | C | drizzle | unknown |
| 14-Apr-19 | 0.2 | E | drizzle | 8888 | na | data not available | unknown |
| 15-Apr-19 | 0 | SW | no rain | 1.3 | N | drizzle | unknown |

0mm = no rain; 0-2.5 mm= drizze; 2,5-10 = moderate rain; 10-50 = heavy rain; >50= storm; 8888 = data not available; na = not available;
C = calm (0m/s)
Rain possibility in the research site was predicted based on the rain state and the wind direction from the climatology stations.
